# Correction: Transparency in conducting and reporting research: A survey of authors, reviewers, and editors across scholarly disciplines

**DOI:** 10.1371/journal.pone.0283443

**Published:** 2023-03-17

**Authors:** The PLOS ONE Staff

## Notice of Republication

This article was republished on March 10, 2023, to address figure errors. The publisher apologizes for the errors. Please download this article again to view the correct version. The originally published, uncorrected article and the republished, corrected articles are provided here for reference.

## Supporting information

S1 File Originally published, uncorrected article. (PDF)

S2 File Republished, corrected article. (PDF)

## References

[1] Malički M, Aalbersberg IJ, Bouter L, Mulligan A, ter Riet G (2023) Transparency in conducting and reporting research: A survey of authors, reviewers, and editors across scholarly disciplines. PLoS ONE 18(3): e0270054. 10.1371/journal.pone.0270054 36888682 PMC9994678

