# Supplementary material for: Transparency in conducting and reporting research: A survey of authors, reviewers, and editors across scholarly disciplines
Source: PLoS One. 2023 Mar 8;18(3):e0270054. doi: 10.1371/journal.pone.0270054 (PMC9994678; doi:10.1371/journal.pone.0270054)
Supplement: S1 Appendix — (DOCX) [file pone.0270054.s001.docx]

**Appendix**

This is an appendix to *Transparency in conducting and reporting research: a survey of authors, editors and peer reviewers* *across scholarly disciplines* by Mario Malički, IJsbrand Jan Aalbersberg, Lex Bouter, Adrian Mulligan, Gerben ter Riet.

Contents

[Respondents’ inquiries 1](#_Toc103941502)

[Response rate 1](#_Toc103941503)

[Generalizability and bias 2](#_Toc103941504)

[Classification of respondents to authors, reviewers, or editors 4](#_Toc103941505)

[Data recoding for reporting and regression analyses 4](#_Toc103941506)

[Respondents’ statistical expertise 6](#_Toc103941507)

[Respondents’ sociodemographic characteristics 7](#_Toc103941508)

[Percentage of respondents with *don’t know* / *not applicable* answers 8](#_Toc103941509)

[Regression Analyses 10](#_Toc103941510)

[References 15](#_Toc103941511)

# Respondents’ inquiries

We received emails from 6 respondents: one mentioning the survey was too long, one asking for results after the survey was done, two asking why they were selected for the survey, and one experiencing difficulty with the questions.

# Response rate

Out of the 100,000 e-mail invites sent, 74,926 were delivered (for 24,691 we received a non-delivery report, 52 addresses were invalid, and 331 respondents opted out of receiving survey invites from the platform we used) and 5,194 opened the survey. However, 781 did not answer a single question (i.e., they just clicked on the survey link), 177 were screened out by the first survey question (as they stated they were neither authors, nor reviewers, nor editors), and 577 answered only the first question (and so we excluded them from the analysis as they shared no opinions on items we were interested in). In total 492 respondents partially completed the survey and 3,167 fully completed it (N=3,659). This leads to a 4.9% response rate (3,659 out of 74,749, with later number representing 74,926 delivered mails minus 177 respondents outside our target audience).

Note: As 177 respondents declared they were not authors, reviewers, or editors, and assuming the same proportion would apply to non-respondents (4% - 177 out of 4,413, with the later number representing those that answered the first question); an additional 2,828 (4% of 70,513 which is 74,926 minus 4,413 that answered the first question) would possibly be outside our target audience. If this were true, our response rate could be calculated as: 3,659 out of 71,921 (74,926 minus 177 and minus 2,828) or 5.1%. However, these percentages are close and are unlikely to affect any generalizability concerns (see below).

# Generalizability and bias

Due to the low response rate (4.9%) and possible selection and non-response bias, our findings are not necessarily generalizable. First, it is likely that editors were more interested to reply to our survey. Out of the 292 invites, which we sent specifically to editors of journals whose *Instruction to Authors* we analysed in our previous study,^1^ 12.6% responded (n=37). In comparison, the response rate for those selected for having a publication in Scopus was 4.9% (3,622 out of 74,457). However, as we did not have information on whether a researcher who published in Scopus is also a reviewer or an editor, we cannot estimate how many of those invited were editors. Another possible indication of higher editors’ interest is a slightly higher percentage of editors responding to open ended questions of our survey. The analysis of open-ended answers will be explored in another publication, but here we report the response percentages: 89% of editors vs 88% of reviewers or 80% of authors elaborated on why they strongly agreed with statements in the survey. Additionally, 15% of editors vs 11% of reviewers or 10% of authors left comments on survey questions, and 27% of editors vs 22% of reviewers or 21% of authors left comments on the overall survey. However, as our study only found slight differences in opinions of editors versus authors and reviewers, this selection bias is unlikely to greatly influence our findings.

Second, in our study we observed significant differences between early survey respondents (those that responded to the initial invite or after first reminder) and late respondents (those that responded after the second reminder), with late respondents having on average more negative attitudes toward transparency (data not shown). Previous research has shown that: 1) the found differences could imply response bias as those that did not respond to our survey might have been even less interested in the topic and harboured even more negative attitudes than those expressed by the late respondents (who in turn had more negative attitudes than the early respondents);^2^ 2) alternatively, non-respondents might have had the same opinions as our late respondents, and therefore finding differences between early and late respondents could indicate that we reached those less willing to answer our survey, making the survey results more likely to be generalizable.^3^ Without contacting those that did not reply (i.e., re-approaching non-respondents) and collecting their answers, we cannot be determine which of the two scenarios is more likely to be true for our survey, nor can we determine how many of our non-respondents refused to participate as they are busy or dislike online surveys, rather than because of their attitudes toward the topic of the survey. Furthermore, to the best of our knowledge, there are no “gold standard” (multiple) imputations methods^4^ or survey weight calculations^5, 6^ for adjusting results based on wave differences nor for adjusting for small response rates found in large online surveys without having information on the characteristics in the target population that make up the selection mechanism. It is possible that due to these reasons, recently published large surveys with less than 10% response rates, did not use any survey weights or non-response imputations.^7, 8^ Nor did recent surveys with smaller sample sizes and higher response rates, with both groups including similarly phrased limitations on their sample’s representativeness.^9^

Classification of respondents to authors, reviewers, or editors

In our first question of the survey, we asked respondents to self-declare if they were authors, reviewers, or editors (multiple roles were allowed). Table A1 describes the respondents’ self-declared roles. For later group comparisons, we classified all those with editor role as editors, all those with reviewer role (minus those that also declared as editors) as reviewers, and the rest as authors.

**Table A1. Respondents’ scholarly roles.**

| **Scholarly Role – n(%)** | **Total Number**  **(n=3,659)** | **For Group Comparison**  **(n=3,656)** |
| --- | --- | --- |
| **Author (A)** | **3,459 (95)** |  |
| Only author | 1,389 (40) | **1389 (38)** |
| Author and Reviewer | 1,678 (49) |  |
| Author and Editor | 27 (1) |  |
| Author, Reviewer and Editor | 365 (11) |  |
| **Reviewer (R)** | **2,209 (60)** |  |
| Only Reviewer | 155 (7) | **1833 (50)** |
| Author and Reviewer | 1,678 (76) |  |
| Reviewer and Editor | 11 (0) |  |
| Author, Reviewer and Editor | 365 (17) |  |
| **Editor (E)** | **434 (12)** | **434 (12)** |
| Only Editor | 31 (7) |  |
| Reviewer and Editor | 11 (3) |  |
| Author and Editor | 27 (6) |  |
| Author, Reviewer and Editor | 365 (84) |  |
| **Not specified** | **3 (0)** | **Excluded** |

# Data recoding for reporting and regression analyses

The following is a list of variables (i.e., survey questions) whose multiple response options were reduced and recoded.

**P1 – Country/Region**

Respondents came from 126 countries (in the text of the manuscript we use the term countries for both). We assigned categories 1 to 4 to countries with most respondents, and 5 to all other countries: 1 – USA, 2 – India, 3 – Italy, 4 – Brazil, 5 – Other.

**P2 - Discipline(s)**

Question P2 of the survey asked respondents: “*In which subject discipline(s) do you work in/publish?”.* Respondents could choose from 28 fields, multidisciplinary category or the “*other*” category where they could write their field(s) using an open text response format. All answers they provided in free text, as well as the 29 choices, were recoded to one of the 6 major categories we also used in our previous study on *Instruction to Authors*: *Arts & Humanities, Health Sciences, Life Sciences, Physical Sciences, Social Sciences,* and *Multidisciplina*ry.^1^ As respondents could select multiple fields, and 2,092 (57%) did so, we applied the following rules during the recoding: 1) if the respondent had chosen more than one field but the majority of chosen fields belonged to one of the 6 major disciplines, they were classified as working predominantly in that discipline (e.g., respondent ID 1718 in the raw data chose 7 disciplines, 4 of which were from *Physical Sciences*, while the other three were one from each of *Health, Life, and Social Sciences,* and so we recoded that respondent as predominantly working in *Physical Sciences);* 2) if the respondent had chosen several fields, but there was an equal number of chosen fields we classified the respondent as working predominantly *Multidisciplina*ry (e.g. respondent ID 778 in the raw data chose 4 disciplines, 2 from *Health Sciences and 2 from Life Sciences*, and so we classified them as *Multidisciplina*ry); 3) if the respondent had chosen a *Multidisciplinary* option out of 29 initial options, they were always classified as *Multidisciplinary, despite the number of other fields they chose* (e.g. respondent ID 2249 in the raw data chose 22 fields, one which was *Multidisciplinary).*

**P3 – Number of authored publications**

We reclassified the original 8 categories, to the following 4: <6, 6 to 25, 26 to 50, and >50.

**P5 - Institution**

We reclassified the original 5 options plus the “other”, to the following 4 categories:

1- University, 2 – Research Institute, 3 – Medical School/Hospital, 4 – Other.

**Q11 – Statistician on last published paper**

Other category was coded and answers aligned where needed to the provided options. The most common answers provided were a co-author, or multiple co-authors, or PhD student(s). Full coding is available on our project’s data repository.^10^

**Additional data cleaning**

Respondent ID 1107 removed their reviewer status before finalizing the survey, so we removed their answers to questions P4 and Q9, and classified them as an author.

# Respondents’ statistical expertise

# Respondents’ sociodemographic characteristics

# Percentage of respondents with *don’t know* / *not applicable* answers

**Table A4. Respondents’ attitudes toward transparency in reporting and conducting research.**

| **Practice (% of respondents with don’t know / not applicable answers)** | **Authors** | **Reviewers** | **Editors** | **Total** |
| --- | --- | --- | --- | --- |
| Authors must appropriately cite all data, analytic methods (program code) and materials used in the study. | 1 | 1 | 1 | 1 |
| Authors must indicate whether the data, analytic methods (program code), and research materials will be made available to any researcher for purposes of reproducing the results or replicating the procedures. | 1 | 1 | 2 | 1 |
| Authors must deposit all data, analytic methods (program code), and research materials to a trusted repository. All exceptions due to legal or ethical reasons must be identified at article submission. | 3 | 2 | 3 | 2 |
| Journals must verify that the findings are replicable using the deposited authors' data and methods of analysis. | 3 | 4 | 3 | 4 |
| Authors must follow appropriate reporting guidelines (e.g. those from www.equator-network.org) for disclosing key aspects of the research design and data analysis. | 6 | 8 | 8 | 8 |
| Journals must check and enforce appropriate reporting guidelines (e.g. those from www.equator-network.org) for disclosing key aspects of the research design and data analysis. | 7 | 9 | 7 | 8 |
| Authors must preregister their study prior to conducting the research. | 6 | 8 | 8 | 7 |
| Journals must check and indicate within the publication that a study had been preregistered prior to the research being conducted. | 8 | 9 | 9 | 9 |
| Authors must include the full data analysis plan in their study preregistration. * | 9 | 11 | 14 | 10 |
| Journals must encourage submission of replication studies, particularly of research they publish. | 5 | 5 | 7 | 5 |
| Journals must employ a two-stage review process for replication studies - in the first stage review the proposal for the replication study, and in the second the full conducted study. * | 9 | 11 | 14 | 11 |

*Chi-square test significant at P<0.05

**Table A5. Respondents’ perceptions toward their work climate. In bold are those where a larger than 5% difference exists between groups.**

| **Practice (%)** | **Authors**  **(n=1,389)** | **Reviewers**  **(n=1,833)** | **Editors**  **(n=434)** | **Total**  **(n=3,656)** |
| --- | --- | --- | --- | --- |
| Due to the pressure to publish, I sacrifice the quality of my publications for quantity. ***** | 2 | 1 | 1 | 1 |
| I am willing to publish studies with null or negative results. | 3 | 3 | 5 | 3 |
| It is difficult to publish studies with null or negative results. | 6 | 6 | 8 | 6 |
| Funders/sponsors interfere in my study design or study reporting. ***** | 13 | 10 | 10 | 11 |
| Unless legal or ethical reasons prevent it, I share my research data with other researchers. | 3 | 3 | 2 | 3 |
| Having access to others' research data benefits/would benefit my own research. | 3 | 2 | 3 | 3 |
| I find it easy to obtain ethics opinions (approvals) for my studies. ***** | 14 | 17 | 13 | 15 |
| Quality of peer review I received for my publications was generally high. ** | 3 | 1 | 1 | 1 |
| Time taken to have my work peer-reviewed has affected my career negatively. ** | 5 | 2 | 3 | 3 |
| Quality of mentoring of young scientists/PhD students in my field is generally high. ** | 8 | 4 | 3 | 5 |
| Quality of publications in my field is generally high. ***** | 2 | 1 | 0 | 1 |
| There is sufficient funding availability for research in my field. ***** | 4 | 2 | 2 | 3 |
| Authors recommending peer reviewers for their research is a good practice. ***** | 3 | 1 | 1 | 2 |

*Chi-square test significant at P<0.05

******Chi-square test significant at P<0.001

**Table A6. Respondents’ perceptions toward their work climate. In bold are those where a larger than 5% difference exists between groups.**

| **Practice (%)** | **Authors** | **Reviewers** | **Editors** | **Total** |
| --- | --- | --- | --- | --- |
| Fabrication or falsification (incl. Image manipulation) ****** | **17** | **14** | **8** | 14 |
| Plagiarism****** | **12** | 8 | **5** | 9 |
| References being omitted (i.e. prior relevant research not being cited) ****** | **8** | 4 | **3** | 5 |
| Publication of corrections (i.e. errata, corrigenda) ****** | **12** | 6 | **5** | 8 |
| Publication of retractions (i.e. study withdrawal) ****** | **20** | **11** | **6** | 13 |
| Undeserved authorship (i.e. guest or gift authorship) ****** | **16** | 10 | **8** | 12 |
| Ghost writing (i.e.  author(s) not being acknowledged) | 23 | 23 | 19 | 22 |
| Undeclared conflict(s) of interest / competing interest(s) ****** | 20 | 18 | 9 | 18 |
| Publication of studies with null or negative results***** | 11 | 7 | 7 | 8 |
| Self-reporting of study limitations****** | **16** | 10 | **6** | 11 |
| Sharing of relevant raw data underlying a research study****** | **15** | 10 | **7** | 11 |
| Use of reporting guidelines for disclosing key aspects of the research design and data analysis***** | 26 | 24 | 18 | 24 |
| Open peer review (i.e. reviewers signing their review reports) ****** | **18** | 7 | **5** | 11 |
| Publishing of authors' versions (non-peer reviewed versions) on pre-print servers ****** | **26** | 15 | **12** | 19 |

*Chi-square test significant at P<0.05

******Chi-square test significant at P<0.001

# Regression Analyses

# References

1. Malicki M, Aalbersberg IJJ, Bouter L, Ter Riet G. Journals' instructions to authors: A cross-sectional study across scientific disciplines. PLOS One. 2019;14(9):e0222157. <https://doi.org/10.1371/journal.pone.0222157>

2. Armstrong JS, Overton TS. Estimating nonresponse bias in mail surveys. Journal of marketing research. 1977;14(3):396-402.

3. Heffetz O, Reeves DB. Difficulty of reaching respondents and nonresponse Bias: Evidence from large government surveys. Review of Economics and Statistics. 2019;101(1):176-91.

4. Kinney SK, Cooney DA. Nonresponse Bias in Sample Surveys. New Directions for Institutional Research. 2019;2019(181):35-46.

5. Groves RM. Nonresponse rates and nonresponse bias in household surveys. Public opinion quarterly. 2006;70(5):646-75.

6. Brasel K, Haider A, Haukoos J. Practical Guide to Survey Research. JAMA surgery. 2020;155(4):351-2.

7. Zotareli V, Souza RT, Cecatti JG. Networks for studies on reproductive and perinatal health: Searching for a consensus. International journal of gynaecology and obstetrics: the official organ of the International Federation of Gynaecology and Obstetrics. 2020;148(3):344-54. <https://doi.org/10.1002/ijgo.13074>

8. Woolston C. Postdoc survey reveals disenchantment with working life. Nature. 2020;587(7834):505-8.

9. Poynton TA, DeFouw ER, Morizio LJ. A systematic review of online response rates in four counseling journals. Journal of Counseling & Development. 2019;97(1):33-42.

10. Malički M, ter Riet G, Bouter LM, Aalbersberg IJJ. Project: Fostering Transparent and Responsible Conduct of Research: What can Journals do? Mendeley Data; 2019. Available from: <http://dx.doi.org/10.17632/53cskwwpdn.6>.
