# Supplementary material for: Transparency in conducting and reporting research: A survey of authors, reviewers, and editors across scholarly disciplines
Source: PLoS One. 2023 Mar 8;18(3):e0270054. doi: 10.1371/journal.pone.0270054 (PMC9994678; doi:10.1371/journal.pone.0270054)
Supplement: Attachment — Submitted filename: PONE-D-22-03115_review.docx [file pone.0270054.s002.docx]

*Transparency in conducting and reporting research: a survey of authors, editors and peer reviewers* *across scholarly disciplines* by Mario Malički, IJsbrand Jan Aalbersberg, Lex Bouter, Adrian Mulligan, Gerben ter Riet.

Hervé Maisonneuve

This article is interesting, of good quality and deserves publication subject to some clarification. The Appendix is worth reading.

Comments on a few points

- Introduction (page 3) is well done and sets out the problem. In the first sentence, it would be useful to clarify that the 3 million articles per year data is an estimate of the STM segment, and that there is no HHS data for the number of articles.
- Methods: Correctly stated, given that the study protocol is available. Perhaps add, if available, the estimated time to answer all questions.
- Results (page 6). The population needs to be detailed a bit more, and it's all in the appendix. It must be said that there were 28 fields for the disciplines, and especially cite the proportion for health sciences, the results of which appear below. Further on, the data analysed concern health sciences (xx%) which could be confused with life sciences (25%)
- No comments on all the tables: they are well done; the statistical tests are in the appendix and this is sufficient; perhaps some comments in the text could be reduced as redundant, but it is not essential
- Discussion (page 13 and following). The discussion needs to be reconsidered to address a few points.
- Would it be useful to compare your 4.9% response rate with E Fong's 10.5% response rate on a sample of 110,000 emails. Why such a difference? I haven't looked closely at the E Fong article, and I don't know if it's relevant. <https://journals.plos.org/plosone/article?id=10.1371/journal.pone.0187394>
- The distinction between authors and reviewers is rather artificial as all reviewers are authors. The comparisons of authors / reviewers / editors are disappointing. Should table A1 be discussed?
- It is a pity that the notion of gender has not been explored. These are points for discussion.
- Sometimes the discussion repeats data from the tables, and this could reduce the length a bit; the conclusion is not useful
- I agree with the sentence on page 13 'Direct comparisons of our results with other surveys are difficult'... but the discussion is just that, when there are issues that deserve discussion:
  - The discussion is mainly focused on researchers and very little on journals and peer review;
  - Wouldn't it make sense to discuss the responsibility of journals? This is a huge topic, but isn't it the major point for improving the system. How can TOP be implemented more quickly? Better recognition of peer review, etc. is essential to develop TOP. What can journals do to implement the TOP guidelines faster?
  - Why not compare the implementation of TOPs, guidelines to the implementation of protocol registration: the ICMJE requested protocol registration in 2004, and almost 20 years later, only prestigious journals require and control this registration.
